# Citrus *p*-Synephrine Improves Energy Homeostasis by Regulating Amino Acid Metabolism in HFD-Induced Mice

**DOI:** 10.3390/nu16020248

**Published:** 2024-01-12

**Authors:** Junying Bai, Xiang Tan, Sheng Tang, Xin Liu, Linzi Shao, Chen Wang, Linhua Huang

**Affiliations:** 1 Citrus Research Institute, Southwest University, Chongqing 400700, China; baijunying@swu.edu.cn (J.B.);; 2 National Citrus Engineering Research Center, Chongqing 400700, China; 3 College of Food Science, Southwest University, Chongqing 400700, China

**Keywords:** citrus, *p*-synephrine, HFD, energy homeostasis, metabolome, amino acid

## Abstract

*p*-Synephrine is a common alkaloid widely distributed in citrus fruits. However, the effects of *p*-synephrine on the metabolic profiles of individuals with energy abnormalities are still unclear. In the study, we investigated the effect of *p*-synephrine on energy homeostasis and metabolic profiles using a high fat diet (HFD)-induced mouse model. We found that *p*-synephrine inhibited the gain in body weight, liver weight and white adipose tissues weight induced by HFD. *p*-Synephrine supplementation also reduced levels of serum total cholesterol (TC), triglyceride (TG) and low-density lipoprotein cholesterol (LDL-C) but not to a statistically significant degree. Histological analysis showed that HFD induced excessive lipid accumulation and glycogen loss in the liver and adipocyte enlargement in perirenal fat tissue, while *p*-synephrine supplementation reversed the changes induced by HFD. Moreover, HFD feeding significantly increased mRNA expression levels of tumor necrosis factor-α (TNF-α) and interleukin-1β (IL-1β) and reduced the mRNA expression level of interleukin-10 (IL-10) compared to the control group, while *p*-synephrine supplementation significantly reversed these HFD-induced changes. Liver and serum metabolomic analysis showed that *p*-synephrine supplementation significantly altered small molecule metabolites in liver and serum in HFD mice and that the changes were closely associated with improvement of energy homeostasis. Notably, amino acid metabolism pathways, both in liver and serum samples, were significantly enriched. Our study suggests that *p*-synephrine improves energy homeostasis probably by regulating amino acid metabolism in HFD mice, which provides a novel insight into the action mechanism of *p*-synephrine modulating energy homeostasis.

## 1. Introduction

The rising prevalence of chronic metabolic diseases including hyperlipidemia, obesity, atherosclerosis and non-alcoholic fatty liver disease has seriously affected people’s health and living standards. These chronic metabolic diseases are often associated with an imbalance between energy consumption and absorption. The prevalence of chronic diseases caused by excessive energy intake has currently reached as high as 25.24% in the world, and it is increasing year by year and shows a younger incidence trend [1]. Several current strategies have been used for the management of energy abnormalities and related chronic metabolic diseases, including diet control, exercise, behavior modification, surgery and medication [2]. Moreover, due to their low toxicity and side effects, natural phytochemical compounds having functional properties of anti-lipogenesis have been used as potential intervention candidates for alleviating chronic metabolic diseases.

Citrus fruits (genus *Citrus* in the *Rutaceae*) are some of the most widely planted types of fruits in the world, currently with an estimated production of more than 150 million tons (FAO statistics, http://www.fao.org/faostat/en/ (accessed on 27 December 2023)). Citrus fruits contain many varieties, including oranges, tangerines, limes, grapefruits, lemons and so on [3]. Citrus fruits are very popular and have received much attention since they contain many kinds of natural phytochemicals such as pectin, flavonoid, alkaloid, carotenoid, polyphenol and limonin and thus exhibit many functional properties, such as anti-inflammation, anti-bacteria, anti-oxidation, and anti-lipogenesis [4]. *p*-Synephrine, as the most abundant alkaloid naturally occurring in citrus fruits, has been found to be widely distributed in sweet and bitter oranges [5]. Previous studies have shown that citrus *p*-synephrine is particularly effective in inhibiting obesity and regulating blood pressure, and it has great potential to be applied in the food industry and medicine field [6]. However, the effects of citrus *p*-synephrine on liver and serum metabolites in individuals with energy disorders remain unclear.

Studies on the in vivo pharmacokinetics of 3H-synephrine showed that total radioactivity in urine after oral and intravenous administration of synephrine was comparable, demonstrating that the synephrines were completely absorbed by the intestine [7]. These intestinal metabolites were then transferred to the liver for further metabolism [7]. And two metabolites *p*-hydroxy-mandelic acid and *p*-hydroxyphenylglycol were detected in the liver after oral intake of *p*-synephrine [8]. These previous findings suggest that *p*-synephrine undergoes extensive biochemical transformation in vivo and may therefore affect the metabolic profiles of the body. Therefore, it is speculated that the regulatory effect of citrus *p*-synephrine on energy homeostasis may be related to small molecule metabolites produced in the liver.

In the present study, high-fat diet (HFD) was given to induce energy abnormalities in a mouse model. Serum cholesterol and fatty acid content were measured to assess the effect of *p*-synephrine on blood lipid levels. Liver fat and glycogen content were visualized to evaluate the effect of *p*-synephrine on lipid and sugar metabolism in the liver. The mRNA expression levels of inflammation-related cytokines in adipose tissue were measured to investigate the effect of *p*-synephrine on HFD-induced inflammatory responses. Liver and serum metabolomes were analyzed to investigate the effect of *p*-synephrine on liver and serum metabolites induced by HFD feeding. Finally, correlation analysis was performed to explore the relationship between the changes in small molecule metabolites and the inhibition of lipogenesis. Our study provides a novel insight into the alleviation of energy disorders by citrus *p*-synephrine.

## 2. Materials and Methods

### 2.1. Animal Experiments

*p*-Synephrine with purity greater than 95% was purchased from Macklin Co., Ltd. (Shanghai, China). Ten-week-old male C57BL/6J mice (20 mice, 20–25 g, SPF) were obtained from Hunan Slake Jingda Experimental Animal Co., Ltd. (Changsha, China) After 1 week of adaptive feeding, all mice were randomly divided into three groups (*n* = 6–7 mice per group): (1) Control group (CON)—gavage with 200 μL sterile normal saline for 8 weeks; (2) HFD group (HFD)—HFD containing 60% kcal% fat (D12492) was given [9] and 200 μL sterile normal saline was intragastrically administered for 8 weeks; and (3) Citrus *p*-synephrine intervention group (HFD+PSY)—HFD was given and *p*-synephrine was diurnally intragastrically administered (30 mg/kg body weight) [10,11]. Body weight and food intake were recorded weekly during the intervention period. After the experiment, all mice were anesthetized by intraperitoneal injection of 1% pentobarbital sodium (dose of 45 mg/kg) and sacrificed for cervical dislocation. The blood samples of mice were collected and centrifuged at 3000× *g* for 30 min to collect the serum for further analysis [12].

### 2.2. Biochemical Analysis of Serum Lipid Level

The levels of total cholesterol (TC), triglyceride (TG), high-density lipoprotein cholesterol (HDL-C) and low-density lipoprotein cholesterol (LDL-C) in the serum were measured by using commercially available enzyme-linked immunosorbent assay (ELISA) kits purchased from Sangon Biological Engineering Technology Co., Ltd. (Shanghai, China). The analysis methods were carried out according to the manufacturer’s instructions.

### 2.3. Histology Examination of Liver and White Adipose Tissue

Histopathological changes of liver and white adipose tissue were analyzed using hematoxylin and eosin (H&E) staining, and hepatocyte glycogen content was visualized using periodic acid-Schiff (PAS) staining, which was performed as previously reported [13,14]. Freshly isolated liver and subcutaneous adipose tissue were rapidly fixed in 4% paraformaldehyde/phosphate buffered saline (PBS) (pH = 7.2), then dehydrated and embedded in paraffin. Subsequently, the tissue samples were cut into vertical serial 5 μm sections using an automatic constant temperature freezing microtome (Leica Microsystems, Wetzlar, Germany). Then, H&E staining and PAS staining were carried out. Finally, stained slides were sealed with resinene after being dehydrated to transparency. All digital section images were then visualized at room temperature by using an inverted light microscope (ZEISS Axio Vert A1, Zeiss, Oberkochen, Germany).

### 2.4. Quantitative Real-Time Polymerase Chain Reaction (qRT-PCR) Analysis of Inflammatory Cytokines

After animal experiments, perirenal fat tissue was collected and stored in liquid nitrogen for extracting total RNA [14]. Total RNA in perirenal fat tissue samples was isolated by using TRIzol reagent (Life Technologies, Carlsbad, CA, USA) and quantified using a Nanodrop Spectrophotometer (Thermo Fisher Scientific, Waltham, MA, USA). One μg of total RNA samples of high-quality (OD260/280 = 1.8–2.2, OD260/230 ≥ 2.0) were used to synthesize cDNAs using the Prime Script RT reagent (Takara, Kusatsu, Japan). qRT-PCR analysis was performed using FastStart Universal SYBR Green Master (ROX) (Vazyme, Nanjing, China) on an ABI 7900 or Step-one RT-PCR system (Applied Biosystems, Waltham, MA, USA). β-Actin was used as an internal control to determine the relative expression of target mRNA in perirenal fat tissues by using the 2^−ΔΔCt^ method. Detailed information on the RT-PCR primer sequences is shown in Table 1.

### 2.5. Non-Targeted Metabolome Analysis of Liver Tissue and Serum

The liver metabolome was analyzed referring to the past literature [15]. The serum metabolome was analyzed according to our previous report [16]. Non-targeted metabolome techniques were used to determine and analyze small molecule metabolites in mice liver and serum. Briefly, mice liver tissues were homogenized at a low temperature with a high-throughput tissue crusher, and then 4 times the volume of acetonitrile-methanol mixed solution was added, and mixed using a whirlpool mixer. For serum samples, 4 times the volume of acetonitrile-methanol mixed solution was directly added, and samples were mixed using a whirlpool mixer. Then, the metabolites in mice liver and serum were extracted, treated with ultrasound for 10 min at 40 kHz in an ice bath, and next placed at −20 °C for 1 h. The sample was centrifuged at 4 °C, 8000× *g* for 15 min, and the supernatant was taken for analysis. At the same time, the supernatant of equal volume of different samples was mixed to make quality control (QC) samples for evaluating the condition of samples and instruments in the analysis process.

Chromatography was performed with an AB SCIEX X500R Q-TOF system and a HSS T3 column (Waters, Milford, MA, USA, 1.8 μm, 100 × 2.1 mm); the injection volume was 5 μL, with a flow rate of 0.35 mL/min. The column temperature was 40 °C. Mobile phase A was acetonitrile with 0.1% formic acid, and mobile phase B was water with 0.1% formic acid. The gradient elution program consisted of 0–6 min (2% A and 98% B), 6–9 min (98% A and 2% B), and 9.1 min (2% A and 98% B). Metabolites were detected on a QTRAP 5500 mass spectrometer simultaneously in positive and negative ion modes. The mass parameters were as follows: ion source at 350 °C, and ion ray voltage at –4500 V. Data were collected, aligned, and normalized using Progenesis QI v2.1 software (Nonlinear Dynamics, Newcastle, UK). The metabolite list only contained *m*/*z* 100–1500. Data analysis and chart plotting were completed by using the online MetaboAnalyst 6.0 analysis tool.

### 2.6. Statistical Analysis

Pearson’s correlation analysis was adopted to evaluate the correlation between energy disorder-related symptoms and significantly differential liver and serum metabolites between groups. Data analysis was performed and charts were made using GraphPad Prism 8.0.2 and R 4.3.0 package. The results were considered statistically significant when *p* < 0.05 between groups.

## 3. Results

### 3.1. Citrus p-Synephrine Reduces Body Weight and Fat Content in HFD-Induced Mice

**Figure 1:**
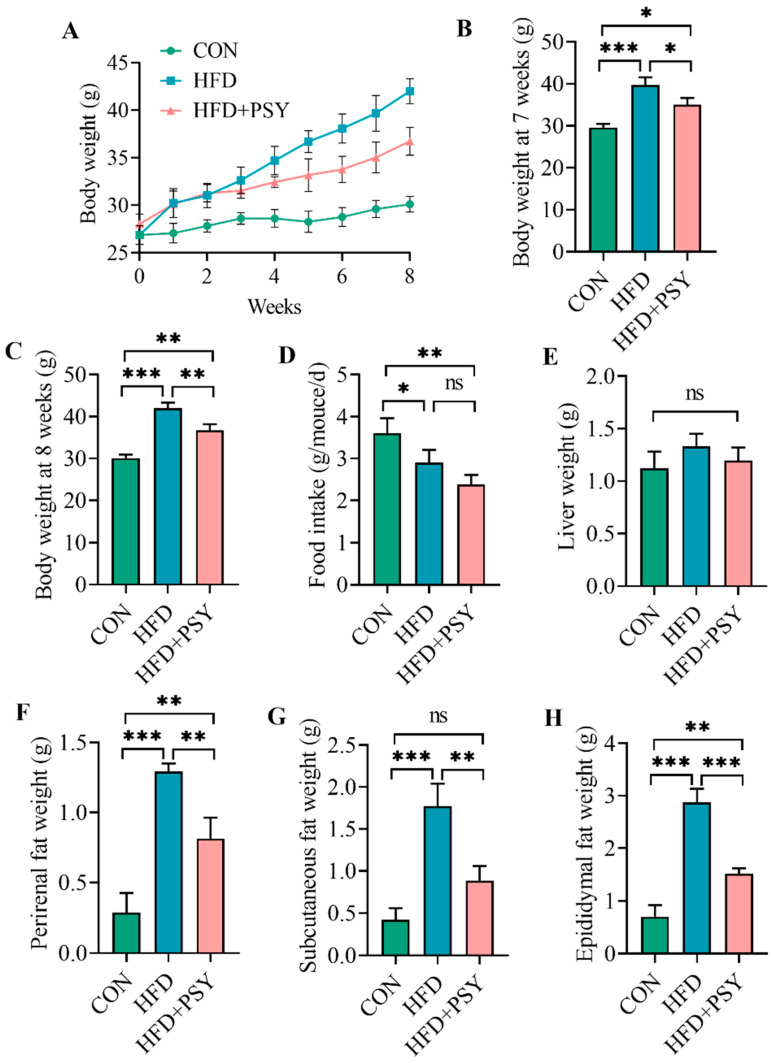
The effect *p*-synephrine supplementation on HFD-induced mice: (**A**) changes in body weight during the 8-week intervention period, (**B**) body weight in the 7th week, (**C**) body weight in the 8th week, (**D**) average daily food intake per mouse, (**E**) liver weight of mice, (**F**) perirenal fat tissue weight of mice, (**G**) subcutaneous fat tissue weight of mice, and (**H**) epididymal fat tissue weight of mice. The results were considered statistically significant when *p* < 0.05 between groups. * *p* < 0.05; ** *p* < 0.01; *** *p* < 0.001; ns, not significant.

Excessive energy intake leads to increased fat content. As shown in Figure 1A, the ingestion of HFD led to a gain in mice body weight compared with the control group in which the mice consumed a normal diet; however, intervention with citrus *p*-synephrine reduced body weight in HFD mice. There was a significant difference in body weight between the CON group, HFD group and HFD+PSY group at 7 weeks and 8 weeks of oral administration of citrus *p*-synephrine (Figure 1B,C). The results of food intake showed that the amount of food intake by control feed mice was higher than that by HFD feed mice and *p*-synephrine supplemented mice, possibly because the energy supply in HFD was greater than that in the control diet. Therefore, the mice in control group consumed more feed to meet their energy needs (Figure 1D). The liver weight of mice increased in the HFD group and decreased in the HFD+PSY group; however, there were no significant differences in these measures among different groups (Figure 1E). By contrast, the weights of perirenal fat, subcutaneous fat and epididymal fat of mice were significantly increased by ingestion of HFD and significantly reduced by gavage of citrus *p*-synephrine (Figure 1F–H). These results suggest that, although long-term HFD intake can lead to excessive fat accumulation in mice, citrus *p*-synephrine intervention is effective in significantly reducing the body weight of mice and inhibiting lipid accumulation in adipose tissue induced by HFD feeding.

### 3.2. Citrus p-Synephrine Reduces Serum Lipid Levels in HFD-Induced Mice

**Figure 2:**
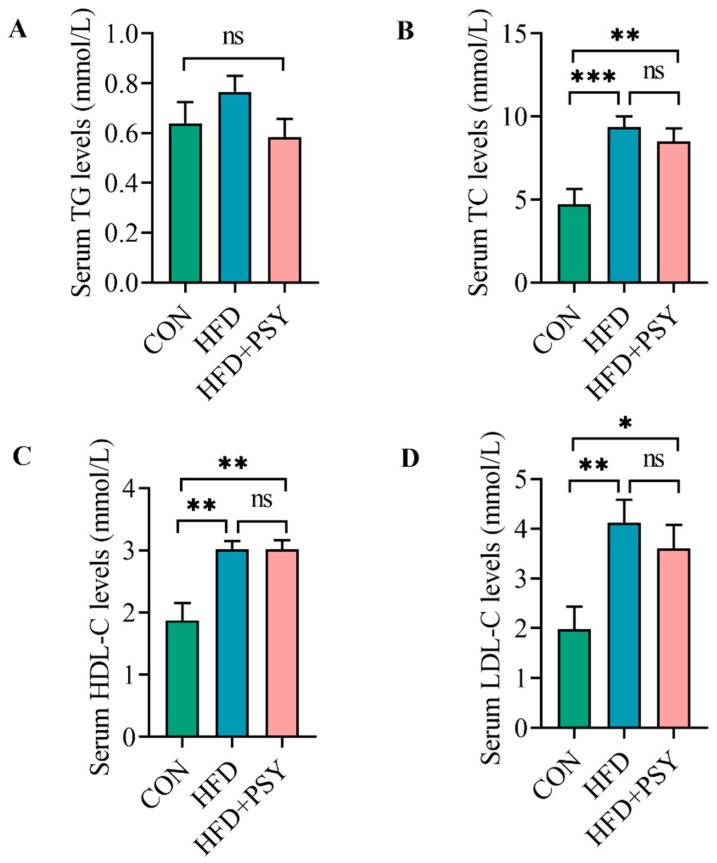
The effect of *p*-synephrine supplementation on serum lipid levels: (**A**) serum TC content, (**B**) TG content, (**C**) HDL-C content and (**D**) LDL-C content. The results were considered statistically significant when *p* < 0.05 between groups. * *p* < 0.05; ** *p* < 0.01; *** *p* < 0.001; ns, not significant.

Serum concentrations of triglycerides and cholesterols can be used as an indicator of lipid levels. In this study, serum TG concentrations in the CON group, HFD group, and HFD+PSY group were 0.64 ± 0.06 mM, 0.77 ± 0.06 mM, and 0.58 ± 0.07 mM, respectively, which showed that the TG levels of different groups were not significantly different (Figure 2A). Serum TC concentrations in the CON group, HFD group, and HFD+PSY group were 4.73 ± 0.91 mM, 9.37 ± 0.64 mM, and 8.50 ± 0.78 mM, respectively (Figure 2B). Compared with the control group, HFD feeding induced significant increases in TC concentration, and *p*-synephrine intervention reduced TC concentrations in HFD mice but not significantly (Figure 2B). Serum HDL-C concentrations in the CON group, HFD group, and HFD+PSY group were 1.87 ± 0.28 mM, 3.02 ± 0.13 mM, and 3.02 ± 0.15 mM, respectively, showing that the gavage of *p*-synephrine did not affect HDL-C levels in the HFD mice (Figure 2C). Serum LDL-C concentrations in the CON group, HFD group, and HFD+PSY group were 1.98 ± 0.46 mM, 4.13 ± 0.45 mM, and 3.61 ± 0.47 mM, respectively, and the trend in changes was consistent with that for serum TC concentration (Figure 2D). These results together suggest that long-term HFD intake can increase serum TG, TC, HDL-C and LDL-C accumulations in mice and that citrus *p*-synephrine supplementation can, to some extent, reduce serum TG, TC and LDL-C levels raised by HFD feeding.

### 3.3. Citrus p-Synephrine Inhibits Energy Abnormalities-Related Symptoms in HFD Mice

**Figure 3:**
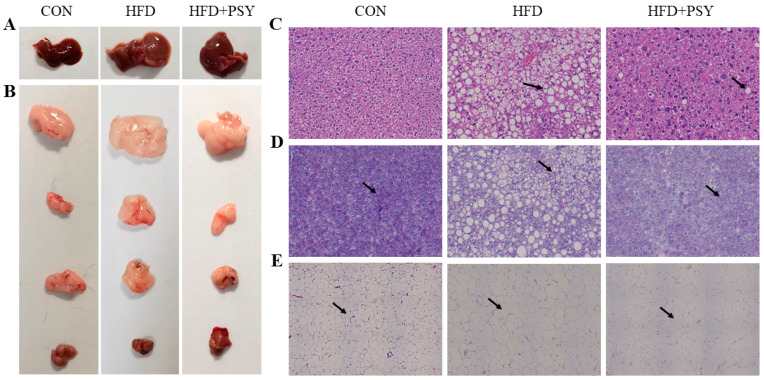
Morphological observation and histopathological analysis of liver and adipose tissues: (**A**) representative images of liver tissue; (**B**) representative images of white adipose tissues and brown fat tissue with epididymal fat, perirenal fat, subcutaneous fat, and brown fat shown in sequence from top to bottom; (**C**) representative images of H&E staining of liver tissue; (**D**) representative images of PAS staining of liver tissue; and (**E**) representative images of H&E staining of subcutaneous fat tissue.

The ingestion of HFD promotes lipid accumulation in liver and adipose tissues, which affects the morphological structure of liver and adipose tissues. As shown in Figure 3A, the liver tissue of the control group was normal and bright red. Liver tissues became significantly larger and turned reddish yellow after ingestion of HFD and returned to their normal red color after intervention with *p*-synephrine. The morphological characteristics of adipose tissues including epididymal fat, perirenal fat, subcutaneous fat and brown adipose tissue are displayed in Figure 3B. HFD significantly increased the volume of white adipose tissues including epididymal fat, perirenal fat and subcutaneous fat and significantly reduced the size of brown fat tissue, whereas *p*-synephrine significantly reduced the size of white adipose tissues and increased the size of brown adipose tissue. The results of H&E staining of liver tissues showed that HFD caused lipid accumulation, and large areas of different-sized lipid droplets were observed in liver tissues, while *p*-synephrine supplementation significantly inhibited liver steatosis in mice (Figure 3C). The results of PAS staining of liver tissues showed that red-stained glycogen content in the liver tissues of HFD mice was lower than that of normal mice, which was reversed by *p*-synephrine (Figure 3D). The results of H&E staining of subcutaneous fat tissues showed that HFD increased the average area of subcutaneous adipocytes, while *p*-synephrine significantly reduced the size of subcutaneous adipocytes in mice (Figure 3E). In general, long-term HFD intake can lead to liver and white adipose tissue steatosis, while intervention with citrus *p*-synephrine can improve the morphological characteristics of liver and adipose tissue in HFD mice.

### 3.4. Citrus p-Synephrine Inhibits HFD-Induced Inflammatory Responses

**Figure 4:**
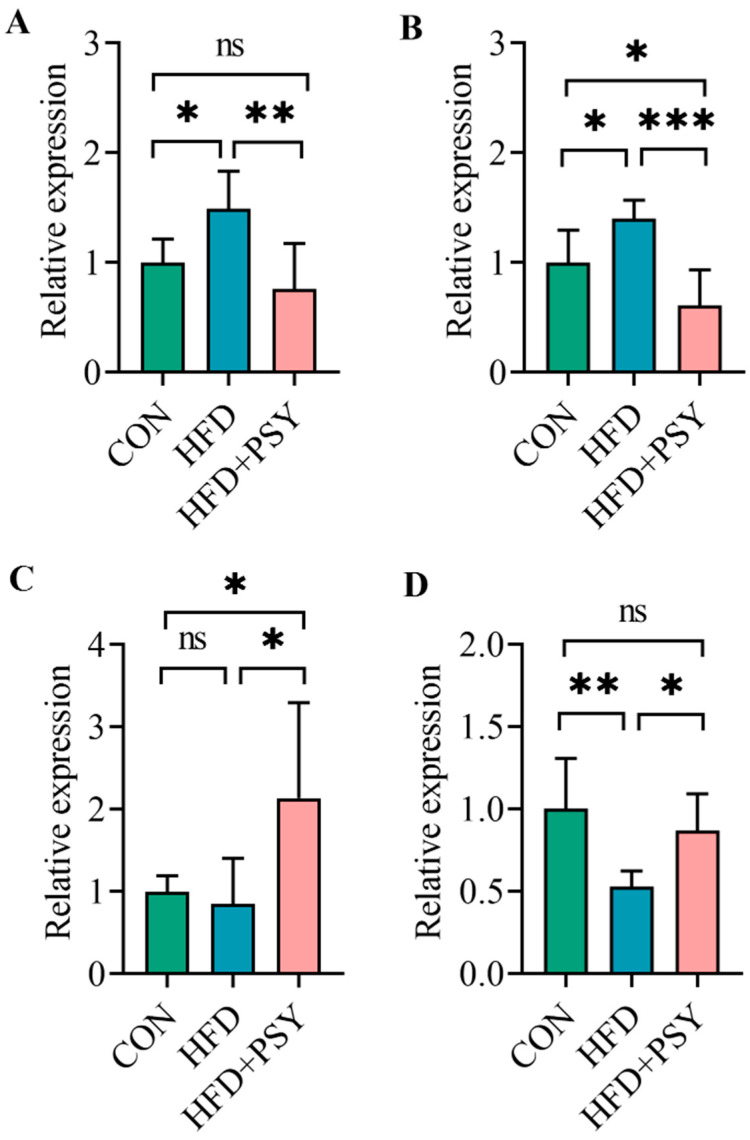
The effect of *p*-synephrine intervention on the mRNA expression levels of inflammatory cytokines: (**A**) TNF-α, (**B**) IL-1β, (**C**) IL-6, and (**D**) IL-10. The results were considered statistically significant when *p* < 0.05 between groups. * *p* < 0.05; ** *p* < 0.01; *** *p* < 0.001; ns, not significant.

The result of inflammatory cytokine mRNA expression levels showed that the ingestion of HFD significantly increased the mRNA expression levels of key pro-inflammatory factors including tumor necrosis factor-α (TNF-α) and interleukin-1β (IL-1β) and significantly reduced the mRNA expression levels of anti-inflammatory factors interleukin-10 (IL-10) in perirenal adipose tissue (Figure 4A,B,D). Oral administration of citrus *p*-synephrine significantly reduced the mRNA expression levels of TNF-α and IL-1β, and increased the mRNA expression levels of IL-10, making them closer to those of the control group (Figure 4A,B,D). The mRNA expression levels of interleukin-6 (IL-6) in perirenal adipose tissue were not significantly changed by HFD treatment, but *p*-synephrine intervention significantly increased the mRNA expression levels of IL-6 (Figure 4C). Taken together, long-term HFD intake could lead to inflammatory responses occurring in white adipose tissue, while citrus *p*-synephrine supplementation remarkably inhibited HFD-induced adipose tissue inflammation.

### 3.5. Citrus p-Synephrine Alters Liver Metabolic Profiles in HFD Mice

**Figure 5:**
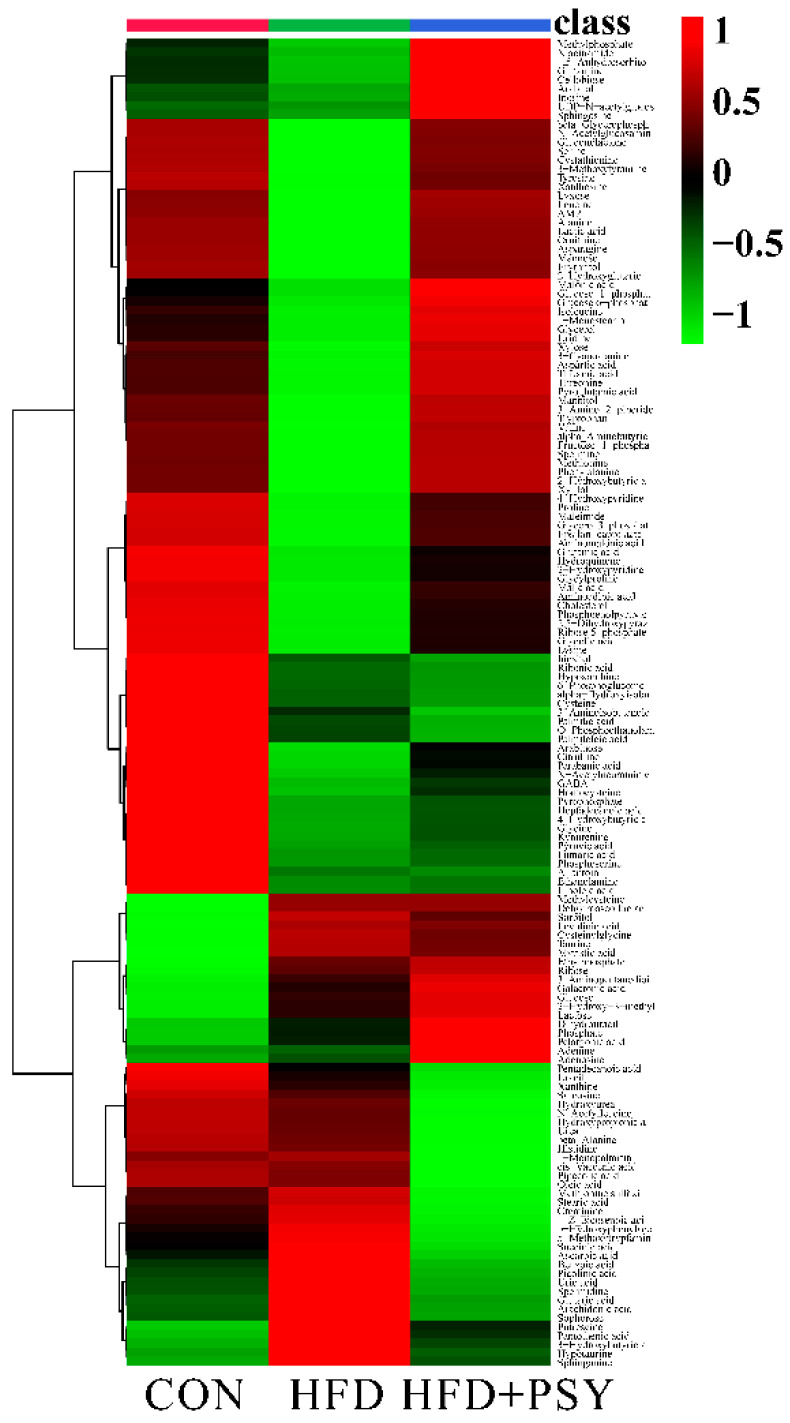
Liver metabolome analysis and heatmap of metabolite distribution among different treatment groups.

**Figure 6:**
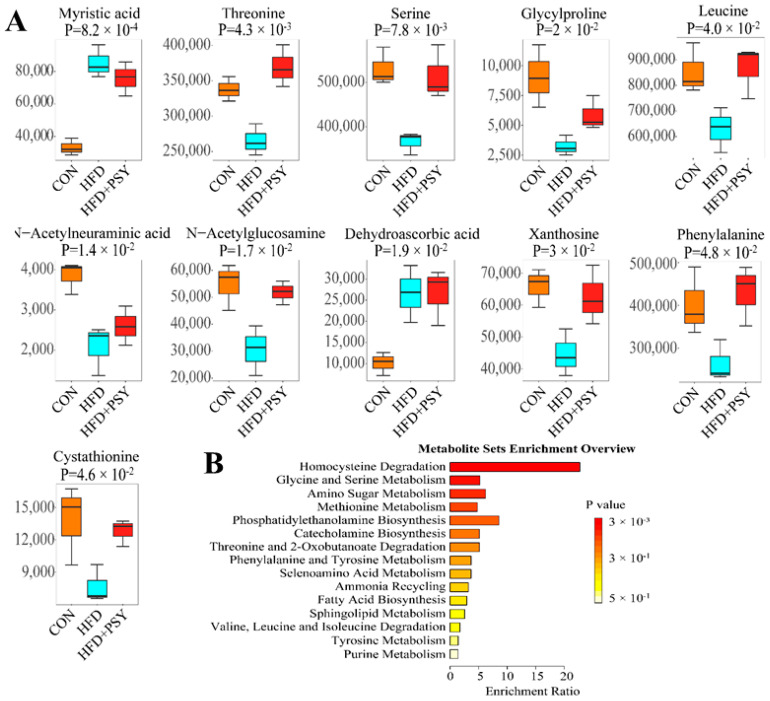
Liver metabolome analysis and representative results: (**A**) metabolites with significant inter-group differences and (**B**) metabolic pathways significantly enriched by differential metabolites.

A total of 149 metabolites in the liver tissues of mice were detected and identified by using non-targeted metabolomics. The distribution of metabolites in different groups of liver samples was visualized by heatmap. Compared with normal control group, HFD feeding significantly changed the metabolite distribution in liver tissues of mice, while oral administration of *p*-synephrine significantly restored liver metabolite profiles of HFD mice, bringing them close to those of control group (Figure 5). Differences in the intensity of metabolites among different treatment groups were compared, and a total of 11 metabolites were found to be significantly different among different groups of mice liver tissues. They were threonine, serine, myristic acid, *N*-acetylneuraminic acid, *N*-acetylglucosamine, dehydroascorbic acid, glycylproline, xanthosine, leucine, cystathionine and phenylalanine (Figure 6A). The ingestion of HFD significantly increased abundances of myristic acid and dehydroascorbic acid, while intervention with *p*-synephrine significantly reduced their abundances in liver tissues of mice. The change in abundances of threonine, serine, *N*-acetylneuraminic acid, *N*-acetylglucosamine, glycylproline, xanthosine, leucine, cystathionine, and phenylalanine exhibited an opposite trend to that of myristic acid and dehydroascorbic acid. The result of enrichment analysis of liver metabolites showing significant differences among different treatment groups revealed the three most significantly related pathways to be homocysteine degradation, glycine and serine metabolism, together with amino sugar metabolism (Figure 6B). In conclusion, long-term HFD intake could lead to significant changes in small molecule metabolites of liver tissues compared with the normal control group, and citrus *p*-synephrine intervention reversed these changes in liver metabolic profiles caused by HFD feeding, which involved the regulation of amino acid metabolism in the liver tissues.

### 3.6. Citrus p-Synephrine Alters Serum Metabolic Profiles in HFD Mice

**Figure 7:**
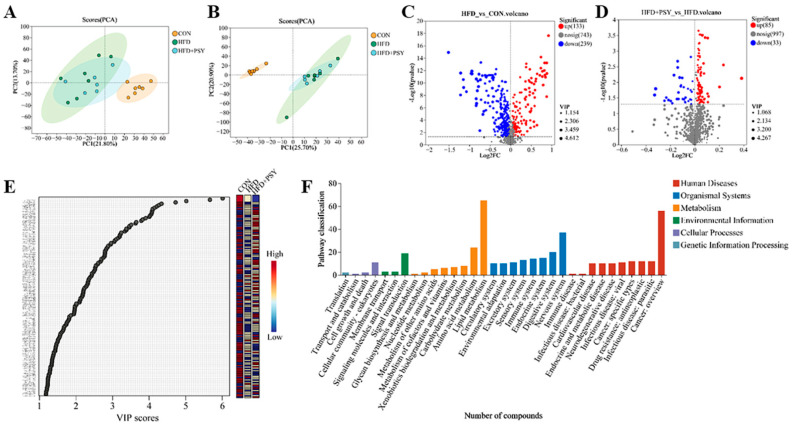
Representative results from serum metabolome analysis: PCA analysis of datasets (**A**) under the positive ion model and (**B**) under the negative ion model; volcano plots from difference analysis (**C**) between the CON group the and HFD group and (**D**) between the HFD group and the *p*-synephrine intervention group; (**E**) VIP values used to reflect differences among multiple groups; and (**F**) metabolic pathways significantly enriched by differential metabolites.

Mice serum was also subjected to non-targeted metabolome examination, and the results of principal components analysis (PCA) of serum metabolites are shown in Figure 6. Under both positive ion and negative ion models, the distribution trend of serum metabolites was obviously different among the three treatment groups (Figure 7A,B). The distribution pattern of mice serum samples in the intervention group receiving *p*-synephrine was between that of the control group and the HFD group, indicating that the intervention with *p*-synephrine restored the changes to mice serum metabolites induced by HFD feeding. The resulting volcano plots show significantly different serum metabolites between the two different treatment groups. Compared with the control group, the ingestion of HFD significantly up-regulated 133 serum metabolites and down-regulated 239 serum metabolites (Figure 7C). After intervention with *p*-synephrine, a total of 85 serum metabolites were up-regulated, and 33 serum metabolites were down-regulated (Figure 7D). Also, the variable importance in projection (VIP) values of multivariate statistical analysis revealed significantly different serum metabolites among all three different treatment groups and metabolites with VIP > 1 being considered as possible biomarkers among groups (Figure 7E). The serum metabolites identified from a combination of volcano plot analysis and multivariate statistical analysis were considered as final metabolite biomarkers reflecting differences among treatment groups. The corresponding metabolic pathways enriched by KEGG functional analysis mainly included lipid metabolism, cancer: overview, nervous system, amino acid metabolism, signal transduction and digestive system (Figure 7F). Taken together, the gavage of citrus *p*-synephrine significantly altered serum metabolic profiles induced by HFD feeding, and the related functional pathways mainly included lipid and amino acid metabolism, signal transduction, nervous and digestive systems, as well as cancer.

### 3.7. Inhibition of Lipogenesis Is Associated with the Regulation of Metabolites

**Figure 8:**
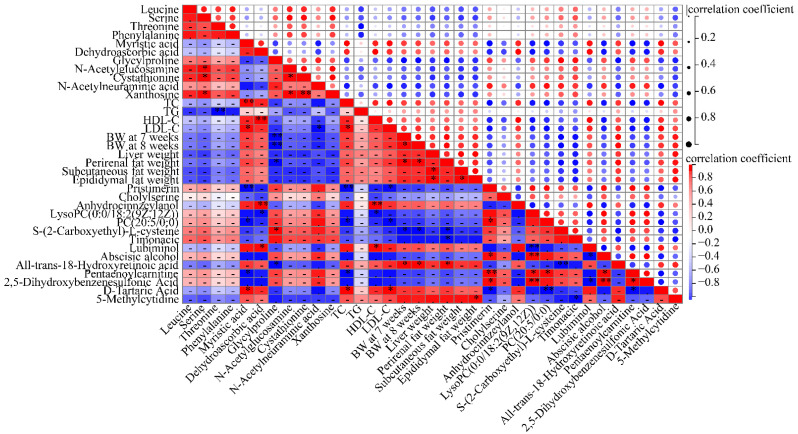
Pearson’s correlation analysis revealed relationships between HFD-induced symptoms and differential metabolites in liver and serum. Pentaenoylcarnitine means (5Z,8Z,10E,14Z,17Z)-12-hydroxyicosa-5,8,10,14,17-pentaenoylcarnitine. * *p* < 0.05; ** *p* < 0.01.

The results of correlation analysis showed the relationship between lipogenesis and significantly different liver and serum metabolites. The results showed that lipid abnormality-related symptoms including TC, TG, HDL-C, LDL-C, body weight and white adipose tissues weight were significantly correlated with liver and serum metabolites (Figure 8). TC content had a positive correlation with the intensity of liver myristic acid and serum D-tartaric acid and a negative correlation with serum pentaenoylcarnitine, PC(20:5/0:0) and pristimerin. TG content had a negative relationship with liver threonine. The level of HDL-C was positively related to liver dehydroascorbic acid, serum lubiminol and anhydrocinnzeylanol and negatively related to serum lysoPC(0:0/18:2(9Z,12Z)). The level of LDL-C was positively related to liver myristic acid and D-tartaric acid and negatively related to liver *N*-acetylneuraminic acid, serum PC(20:5/0:0) and pristimerin. Body weight of mice at 7 and 8 weeks was negatively related to liver glycylproline and serum S-(2-carboxyethyl)-L-cysteine and positively related to all-trans-18-hydroxyretinoic acid. The contents of white adipose tissues including perirenal fat, subcutaneous fat and epididymal fat showed a negative correlation with liver glycylproline, serum S-(2-carboxyethyl)-L-cysteine and timonacic and showed a positive correlation with serum all-trans-18-hydroxyretinoic acid and 5-methylcytidine. Some significant correlations were also found between liver metabolites and serum metabolites, which indicated a probable causality between liver metabolites and serum metabolites. Taken together, these findings suggest that citrus *p*-synephrine alleviates HFD-induced energy disorders, which are mediated by liver and serum metabolite responses to *p*-synephrine.

## 4. Discussion

In the present study, we first evaluated the effect of citrus *p*-synephrine on the phenotype of mice with energy disorders induced by HFD feeding and found that intervention with *p*-synephrine for 8 weeks significantly reduced the gain in body weight, liver weight and white adipose tissue weight. Next, we determined the effect of *p*-synephrine on serum lipid levels and histomorphologies of liver and adipose tissue and found that *p*-synephrine supplementation reduced levels of serum TC, TG, and LDL-C; inhibited lipid accumulation in the liver and adipocyte expansion in white adipose tissue; and increased the glycogen content of liver tissue. We also measured the effect of *p*-synephrine on HFD-induced inflammatory responses of white adipose tissues and found that *p*-synephrine supplementation significantly reduced mRNA expression levels of pro-inflammatory cytokines TNF-α and IL-1β and increased mRNA expression levels of anti-inflammatory cytokines IL-10 in HFD-induced subcutaneous adipose tissue. We then performed liver and serum metabolome analysis and found that *p*-synephrine intervention significantly altered liver and serum metabolic profiles induced by HFD feeding and significantly affected amino acid metabolism pathways in the liver and serum. Finally, Pearson’s correlation analysis revealed a correlation between the alteration in liver and serum metabolites and the alleviation of energy disorders.

Energy abnormalities are major causes of hyperlipidemia, obesity, diabetes, fatty liver disease, cardiovascular disease, etc., which have become the number one killer of human health. Previous studies have shown that *p*-synephrine supplementation can increase energy expenditure and fat oxidation [6,17], mainly through the stimulation of β-adrenergic receptors, with *p*-synephrine being regarded as a naturally occurring β-adrenergic receptor agonist [18]. No studies have reported on the effects of *p*-synephrine on small molecule metabolites in liver and serum. *p*-Synephrine has been demonstrated to be fully absorbed in the small intestine, with its intestinal metabolites finally being completely metabolized in the liver [7,8]. Therefore, there is a need to perform metabolome analysis of liver tissue and serum to investigate whether the alleviation of energy disorders by *p*-synephrine is associated with small molecule metabolites in the liver and serum. We confirmed that *p*-synephrine can improve HFD-induced energy disorders and that the effect is correlated with liver and serum metabolites, which probably provide a novel strategy for targeting small molecule metabolites in vivo to improve energy homeostasis.

Excessive energy intake easily leads to lipid accumulation in the serum, and high blood lipid levels are particularly a risk factor for cardiovascular disease. The levels of serum TC, TG and LDL-C were positively related to energy intake, while serum HDL-C was negatively related to energy intake [19,20]. We found that HFD feeding resulted in elevation of serum TC and significant elevation of serum TG and LDL-C, while *p*-synephrine supplementation obviously reduced the levels of TC, TG and LDL-C, which is consistent with previous studies and proves the effectiveness of *p*-synephrine in lowering blood lipid levels. No significant differences were observed in the effect of lowering serum lipid levels, perhaps because the intervention period was only 8 weeks and relatively short. Moreover, excessive energy intake also could lead to increased lipid storage in liver and adipose tissue overload, consequently causing ectopic lipid depositions and fatty liver [21]. The results of H&E staining proved that HFD feeding caused lipid depositions in liver and adipocyte enlargement in subcutaneous fat tissue, whereas *p*-synephrine supplementation impaired these changes induced by HFD. We found that glycogen content was lowered in the livers of mice treated with HFD and increased in the livers of mice treated with *p*-synephrine, indicating that HFD induced liver steatosis and thus the liver’s ability to transform and store glycogen was reduced, while *p*-synephrine supplementation reversed these changes.

Long-term consumption of HFD contributes to metabolic diseases often along with the development of chronic inflammation and prolonged imbalance of oxidative stress in the body. White adipose tissue is the primary site of energy storage and metabolism in mammals. Dilation, dysfunction, and inflammation of white adipose tissue are hallmarks of obesity and play a key role in the development of highly prevalent lipid abnormality-related diseases such as insulin resistance, atherosclerosis and non-alcoholic fatty liver disease [22]. In the present study, we found that HFD feeding significantly increased mRNA expression levels of pro-inflammatory factors TNF-α and IL-1β and decreased anti-inflammatory factors IL-10 in perirenal adipose tissue, which conforms to previous reports. Supplementation with citrus *p*-synephrine significantly decreased mRNA expression levels of pro-inflammatory factors and increased anti-inflammatory factors in perirenal adipose tissue, which suggests that *p*-synephrine not only inhibits HFD-induced weight gain but also reduces HFD-induced adipose tissue inflammation. This is the first report regarding the inhibitory effects of *p*-synephrine on HFD-related metabolic inflammation. The changes in IL-6 expression level in different treatment groups were puzzling, which might be attributed to the multifaceted nature of IL-6. Previous studies have pointed out that IL-6 is a double-edged sword that exhibits both pro- and anti-inflammatory effects [23].

In recent years, the development of metabolomics has sped up the understanding of global metabolic characteristics, contributing to the identification of metabolic biomarkers and elucidation of metabolic mechanisms related to energy metabolism imbalance [24]. In the present study, we performed metabolome analysis on liver and serum metabolites and found that HFD feeding and *p*-synephrine supplementation both significantly altered small molecule metabolites in the liver and serum. The connection between some metabolites such as myristic acid, *N*-acetylneuraminic acid or phenylalanine and energy homeostasis has previously been established. HFD consumption significantly increased the level of myristic acid in the liver of mice [25]. The ingestion of *N*-acetylneuraminic acid attenuated HFD-induced inflammation and oxidative stress, and attenuated hypercoagulation in HFD-induced hyperlipidemic rats [26,27]. Phenylalanine biosynthesis played a role in improving HFD-induced obesity treated with *Sporisorium reilianum* polysaccharide and metabolic disorders of type 2 diabetic mellitus combined with non-alcoholic fatty liver disease in mice treated with Zuogui-Jiangtang-Qinggan-Fang [28,29]. These previous findings are consistent with our results.

We found that amino acids were the largest group of metabolites with significant differences between groups, and amino acid metabolism pathways were also significantly enriched in liver tissue, which suggests that *p*-synephrine intervention significantly affects the liver amino acid metabolism pathway in HFD-induced mice. Serum metabolomic analysis showed that *p*-synephrine intervention also significantly alters the serum amino acid metabolism pathway in HFD mice. Correlation analysis revealed that the alleviating effect of *p*-synephrine on energy abnormalities is closely associated with the regulation of liver and serum metabolites. These findings together suggest that citrus *p*-synephrine likely improves energy homeostasis by regulating amino acid metabolism pathways in the body. The relationship between amino acid metabolism and energy homeostasis has been previously established [30,31,32]. Specific amino acids have also been shown to play a role in energy metabolism. For example, dietary supplementation with L-arginine can reduce fat accumulation in viscera through a change in matrix metalloproteinase activity and amelioration of insulin sensitivity in rats fed a HFD [33]. Intraperitoneal injection of D-serine inhibited HFD intake and preferences in male mice [34]. Our study found that the improvement of energy homeostasis by *p*-synephrine is closely associated with amino acid metabolism, which provides a novel insight into the action mechanism of *p*-synephrine in alleviating energy abnormalities.

## 5. Conclusions

In conclusion, we found that citrus *p*-synephrine supplementation can not only reduce HFD-induced energy abnormality-related symptoms but also inhibit HFD-induced adipose tissue inflammatory responses and demonstrated that the alleviating effect of *p*-synephrine on HFD-induced energy disorders is closely associated with changes in liver and serum metabolites and especially the role of amino acid metabolism. Our study will broaden the application of citrus *p*-synephrine as a functional component and provides a novel strategy for targeting liver and serum metabolites to improve energy homeostasis. Future studies could further explore the detailed action mechanisms by which citrus *p*-synephrine improves energy homeostasis by regulating amino acid metabolism.

## Figures and Tables

**Table 1:** Detailed information on primer sequences.

Gene	Sense Sequence (5′→3′)	Antisense Sequence (5′→3′)
β-actin	GGCTGTATTCCCCTCCATCG	CCAGTTGGTAACAATGCCATGT
IL-6	CCACTTCACAAGTCGGAGGCTTA	GCAAGTGCATCATCGTTGTTCATAC
IL-10	GCTCTTACTGACTGGCATGAG	CGCAGCTCTAGGAGCATGTG
TNF-α	ACACCGAGATTTCCTTCAAACTG	CCATCTAGGGTTATGATGCTCTTCA
IL-1β	GGGCCTCAAAGGAAAGAATC	TACCAGTTGGGGAACTCTGC

## Data Availability

The data presented in this study are available on request from the corresponding author.

## Abbreviations

HFD—high fat diet; TC—total cholesterol; TG—triglyceride; LDL-C—low-density lipoprotein cholesterol; HDL-C—high-density lipoprotein cholesterol; CON—control group; ELISA—enzyme-linked immunosorbent assay; H&E—hematoxylin and eosin; PAS—periodic acid-Schiff; PBS—phosphate buffered saline; RT-PCR—real-time polymerase chain reaction; qRT-PCR—quantitative real-time polymerase chain reaction; QC—quality control; TNF-α—tumor necrosis factor-α; IL-1β—interleukin-1β; IL-6—interleukin-6; IL-10—interleukin-10; PCA—principal component analysis; VIP—variable importance in projection.
